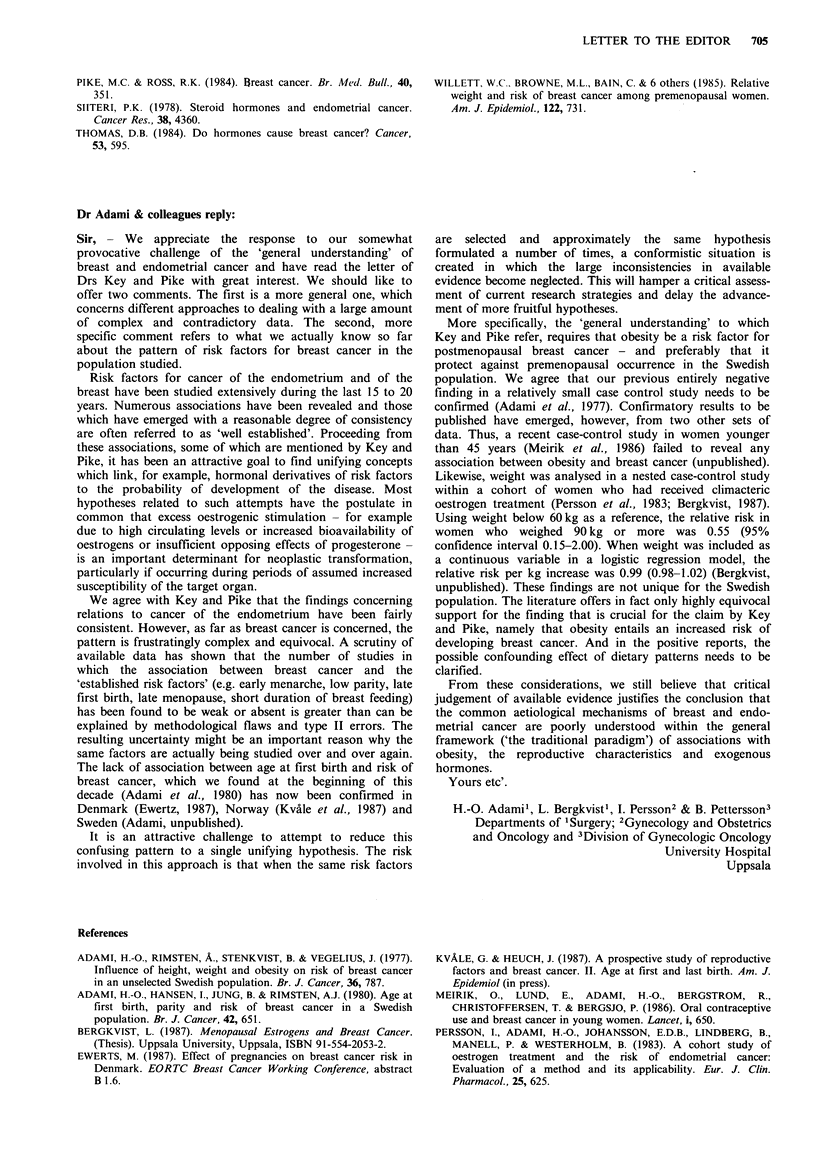# Dr Adami & colleagues reply

**Published:** 1987-11

**Authors:** H.-O. Adami, L. Bergkvist, I. Persson, B. Petterson


**Dr Adami & colleagues reply:**

**Sir,** – We appreciate the response to our somewhat provocative challenge of the 'general understanding' of breast and endometrial cancer and have read the letter of Drs Key and Pike with great interest. We should like to offer two comments. The first is a more general one, which concerns different approaches to dealing with a large amount of complex and contradictory data. The second, more specific comment refers to what we actually know so far about the pattern of risk factors for breast cancer in the population studied.

Risk factors for cancer of the endometrium and of the breast have been studied extensively during the last 15 to 20 years. Numerous associations have been revealed and those which have emerged with a reasonable degree of consistency are often referred to as 'well established'. Proceeding from these associations, some of which are mentioned by Key and Pike, it has been an attractive goal to find unifying concepts which link, for example, hormonal derivatives of risk factors to the probability of development of the disease. Most hypotheses related to such attempts have the postulate in common that excess oestrogenic stimulation – for example due to high circulating levels or increased bioavailability of oestrogens or insufficient opposing effects of progesterone – is an important determinant for neoplastic transformation, particularly if occurring during periods of assumed increased susceptibility of the target organ.

We agree with Key and Pike that the findings concerning relations to cancer of the endometrium have been fairly consistent. However, as far as breast cancer is concerned, the pattern is frustratingly complex and equivocal. A scrutiny of available data has shown that the number of studies in which the association between breast cancer and the 'established risk factors' (e.g. early menarche, low parity, late first birth, late menopause, short duration of breast feeding) has been found to be weak or absent is greater than can be explained by methodological flaws and type II errors. The resulting uncertainty might be an important reason why the same factors are actually being studied over and over again. The lack of association between age at first birth and risk of breast cancer, which we found at the beginning of this decade (Adami *et al.*, 1980) has now been confirmed in Denmark (Ewertz, 1987), Norway (Kvåle *et al.*, 1987) and Sweden (Adami, unpublished).

It is an attractive challenge to attempt to reduce this confusing pattern to a single unifying hypothesis. The risk involved in this approach is that when the same risk factors are selected and approximately the same hypothesis formulated a number of times, a conformistic situation is created in which the large inconsistencies in available evidence become neglected. This will hamper a critical assessment of current research strategies and delay the advancement of more fruitful hypotheses.

More specifically, the 'general understanding' to which Key and Pike refer, requires that obesity be a risk factor for postmenopausal breast cancer – and preferably that it protect against premenopausal occurrence in the Swedish population. We agree that our previous entirely negative finding in a relatively small case control study needs to be confirmed (Adami *et al.*, 1977). Confirmatory results to be published have emerged, however, from two other sets of data. Thus, a recent case-control study in women younger than 45 years (Meirik *et al.*, 1986) failed to reveal any association between obesity and breast cancer (unpublished). Likewise, weight was analysed in a nested case-control study within a cohort of women who had received climacteric oestrogen treatment (Persson *et al.*, 1983; Bergkvist, 1987). Using weight below 60 kg as a reference, the relative risk in women who weighed 90 kg or more was 0.55 (95% confidence interval 0.15–2.00). When weight was included as a continuous variable in a logistic regression model, the relative risk per kg increase was 0.99 (0.98–1.02) (Bergkvist, unpublished). These findings are not unique for the Swedish population. The literature offers in fact only highly equivocal support for the finding that is crucial for the claim by Key and Pike, namely that obesity entails an increased risk of developing breast cancer. And in the positive reports, the possible confounding effect of dietary patterns needs to be clarified.

From these considerations, we still believe that critical judgement of available evidence justifies the conclusion that the common aetiological mechanisms of breast and endometrial cancer are poorly understood within the general framework ('the traditional paradigm') of associations with obesity, the reproductive characteristics and exogenous hormones.

Yours etc'.

H.-O. Adami[1], L. Bergkvist[1], I. Persson[2] & B. Pettersson[3]
Departments of [1]Surgery; [2]Gynecology and Obstetrics and Oncology and [3]Division of Gynecologic Oncology
University Hospital
Uppsala

**References**

ADAMI, H.-O., RIMSTEN, Å., STENKVIST, B. & VEGELIUS, J. (1977). Influence of height, weight and obesity on risk of breast cancer in an unselected Swedish population. *Br. J. Cancer*, **36**, 787.

ADAMI, H.-O., HANSEN, I., JUNG, B. & RIMSTEN, A.J. (1980). Age at first birth, parity and risk of breast cancer in a Swedish population. *Br. J. Cancer*, **42**, 651.

BERGKVIST, L. (1987). *Menopausal Estrogens and Breast Cancer.* (Thesis). Uppsala University, Uppsala, ISBN 91-554-2053-2.

EWERTS, M. (1987). Effect of pregnancies on breast cancer risk in Denmark. *EORTC Breast Cancer Working Conference*, abstract B 1.6.

KVÅLE, G. & HEUCH, J. (1987). A prospective study of reproductive factors and breast cancer. II. Age at first and last birth. *Am. J. Epidemiol* (in press).

MEIRIK, O., LUND, E., ADAMI, H.-O., BERGSTROM, R., CHRISTOFFERSEN, T. & BERGSJO, P. (1986). Oral contraceptive use and breast cancer in young women. *Lancet*, **i**, 650.

PERSSON, I., ADAMI, H.-O., JOHANSSON, E.D.B., LINDBERG, B., MANELL, P. & WESTERHOLM, B. (1983). A cohort study of oestrogen treatment and the risk of endometrial cancer: Evaluation of a method and its applicability. *Eur. J. Clin. Pharmacol.*, **25**, 625.